# A review of clinical trials of cetuximab combined with radiotherapy for non-small cell lung cancer

**DOI:** 10.1186/1748-717X-7-3

**Published:** 2012-01-11

**Authors:** Carsten Nieder, Adam Pawinski, Astrid Dalhaug, Nicolaus Andratschke

**Affiliations:** 1 Department of Oncology and Palliative Medicine, Nordland Hospital, Bodø, Norway; 2 Institute of Clinical Medicine, Faculty of Health Sciences, University of Tromsø, Tromsø, Norway; 3 Department of Radiation Oncology, Klinikum rechts der Isar der Technischen Universität München, Munich, Germany

**Keywords:** non-small cell lung cancer, radiotherapy, cetuximab, chemoradiation

## Abstract

Treatment of non-small cell lung cancer (NSCLC) is challenging in many ways. One of the problems is disappointing local control rates in larger volume disease. Moreover, the likelihood of both nodal and distant spread increases with primary tumour (T-) stage. Many patients are elderly and have considerable comorbidity. Therefore, aggressive combined modality treatment might be contraindicated or poorly tolerated. In many cases with larger tumour volume, sufficiently high radiation doses can not be administered because the tolerance of surrounding normal tissues must be respected. Under such circumstances, simultaneous administration of radiosensitizing agents, which increase tumour cell kill, might improve the therapeutic ratio. If such agents have a favourable toxicity profile, even elderly patients might tolerate concomitant treatment. Based on sound preclinical evidence, several relatively small studies have examined radiotherapy (RT) with cetuximab in stage III NSCLC. Three different strategies were pursued: 1) RT plus cetuximab (2 studies), 2) induction chemotherapy followed by RT plus cetuximab (2 studies) and 3) concomitant RT and chemotherapy plus cetuximab (2 studies). Radiation doses were limited to 60-70 Gy. As a result of study design, in particular lack of randomised comparison between cetuximab and no cetuximab, the efficacy results are difficult to interpret. However, strategy 1) and 3) appear more promising than induction chemotherapy followed by RT and cetuximab. Toxicity and adverse events were more common when concomitant chemotherapy was given. Nevertheless, combined treatment appears feasible. The role of consolidation cetuximab after RT is uncertain. A large randomised phase III study of combined RT, chemotherapy and cetuximab has been initiated.

## Introduction

Non-small cell lung cancer (NSCLC) is among the leading causes of cancer death in the western world and increasing in many other countries. Survival of patients with locoregionally advanced disease (stage III) and metastatic disease has remained disappointing despite some gradual improvement [1,2]. Patients with stage III disease differ with regard to primary tumour volume and proximity/infiltration to surrounding structures, extent of lymphatic spread, cancer biology, and host factors such as age, cardiopulmonary function and other comorbidity [3]. Treatment recommendations have to take into account these differences and stratify patients according to technical resectability, ability to tolerate high-dose radiotherapy and chemotherapy, and many more.

Many patients with inoperable stage III disease are candidates for combined modality chemo- and radiotherapy (RT). While concomitant administration might improve survival, parallel increases of toxicity have also been observed [1,4]. The dilemma of simultaneous increases in efficacy and toxicity becomes even more difficult in elderly patients and those with considerable pretreatment weight loss, reduced performance status and comorbidity. Incorporation of novel agents might be advantageous in several ways. It might allow for administration of combined modality treatment in patients who are not candidates for established chemoradiation regimens and where RT alone results in unsatisfactory outcomes. Moreover, certain agents might be added on top of chemoradiation with the aim of further improving treatment results in patients who can tolerate aggressive approaches.

The theoretical solution of simply increasing radiation doses to high biologically effective doses (BED), ideally above the threshold of 100 Gy in 2-Gy fractions, which has been suggested by several groups [5-8], is hampered by the tolerance of surrounding normal tissues that must be respected if a favourable therapeutic ratio is to be maintained. Under such circumstances, simultaneous administration of radiosensitizing agents that increase tumour cell kill might improve the therapeutic ratio, provided these agents do not sensitize critical normal tissues in the same fashion. Moreover, radiation dose escalation does not address the issue of distant or out-of-field relapses. Based on the fact that the epidermal growth factor receptor (EGFR) is often over-expressed or mutated in NSCLC, the impact of such changes on cellular responses to ionising radiation has been explored [9-11]. Several drugs interfering with the EGFR signalling pathway have been developed, e.g. cetuximab, a human-murine chimeric IgG1 monoclonal antibody that binds to the extracellular region of the EGFR. Under experimental laboratory conditions in animal models, cetuximab increases tumour radiocurability (fractionated and single dose irradiation) [12,13]. Clinically, this effect has been confirmed in head and neck cancer (phase III data with fractionated irradiation) [14]. Recently, initial clinical trials of cetuximab and RT for NSCLC have been completed, which are reviewed here in order to guide the development of the next generation of larger prospective studies. The data included in this review were identified by comprehensive searches of the PubMed database with combinations of the keywords "NSCLC, RT, cetuximab, EFGR" (last access August 15, 2011) and the reference lists of articles.

### Cetuximab in patients who are not candidates for chemoradiation

As summarized in Tables 1 and 2, two different phase II trials have studied combined RT and cetuximab without any chemotherapy [15,16]. In the US trial, cetuximab was given concomitant to 60 Gy RT [15]. In the German trial, intensity-modulated RT to a total dose of 66 Gy was combined with concomitant and 13 weekly consolidation cycles of cetuximab [16]. Another important difference is that positron emission tomography (PET) was mandatory in the German trial, which also included patients with slightly more favourable baseline prognostic factors (some stage II patients, no weight loss > 5%, younger median age). Median survival and response rates were higher in the German trial. However, such differences might result from treatment itself or inclusion of prognostically better patients. Mucosal and pulmonary toxicities occurred at the expected frequencies in patients irradiated for stage III NSCLC (elective nodal irradiation (ENI) was part of the treatment concept) but overall compliance and tolerability suggest that concomitant cetuximab and standard dose RT (60-66 Gy) is feasible even in patients unfit for chemoradiation. The median survival of 15.1 and 19.6 months respectively compares favourably with trials of comparable RT alone, e.g. the series of 127 patients reported by Jeremic et al. who found a median survival of 12 months [17], and the series of 106 patients reported by Wang et al. where median survival was 7.4 months [18]. However, such inter-study comparison might be hampered by several sources of bias (patient selection, improved staging, improved RT delivery etc.) and can not prove the superiority of combined treatment. Randomised comparison of RT alone and RT with cetuximab is still necessary. It would also be interesting to study whether irradiation to 60-66 Gy plus cetuximab is better than dose-escalated RT without cetuximab, given the high cost resulting from drug treatment.

**Table 1:** Study design and efficacy

Reference	Study type	Patients	Other criteria	RT schedule	Systemic therapy	Follow-up	Results
Jensen et al. [16]	Single institution phase II, Germany, n = 30	Not candidates for concomitant chemoradiation (or refused), KPS at least 70, one of two trials with mandatory PET	Stage IIIA or B, no malignant pleural effusion, FEV1 ≥ 1.5 l or 40% of norm value, weight loss < 5%	Only IMRT trial, 66 Gy in 33 daily fractions of 2 Gy, ENI to 50 Gy (or 40 depending on lung dose, target volume NR)	Cetuximab SR followed by 13 weekly consolidation cycles	Median 19 mo	Median OS 19.6 mo, median PFS 8.5 mo, 63% PR, no CR, patterns of failure NR, survival not influenced by histology
Jatoi et al. [15]	Multi-centre phase II, US, n = 58	Not candidates for concomitant chemoradiation, either age ≥ 65 years with ECOG 0-2 or younger but ECOG 2	Stage III A or B, no pleural effusion, FEV1 ≥ 1 l, haemoglobin ≥ 9 g/dl, V20 not exceeding 40%	60 Gy in 30 daily fractions of 2 Gy, ENI to ipsilateral hilar and mediastinal nodes (44 Gy)	Cetuximab SR	Median 17 mo	Median OS 15.1 mo, median PFS 7.2 mo, 26% PR, no CR, patterns of failure and impact of histology NR
Hallqvist et al. [19]	Multi-centre phase II, Sweden, n = 71	Medically inoperable or unresectable, WHO 0-1	Stage IIIA or B, no pleural effusion with positive cytology, FEV1 ≥ 1 l or 40% of expected volume	68 Gy in 34 daily fractions of 2 Gy, no ENI	2 cycles of induction cisplatin/docetaxel, cetuximab SR starting one week before RT	Median 39 mo	Median OS 17 mo, PFS NR, 16% PR and 7% CR at 12 months (NR at earlier time points), patterns of failure: 31% distant only, 23% local only, 7% regional only, 11% combinations of these, survival not influenced by histology
Hughes et al. [20]	Dual centre phase II, UK, n = 12	Inoperable, WHO 0-1	Stage IIIA or B, no pleural effusion	64 Gy in 32 fractions of 2 Gy, in 4 cases ENI to ipsilateral hilar and mediastinal nodes (50 Gy)	Up to 4 cycles (median 3) of platinum-based induction CTx, cetuximab SR starting one week before RT	Median 10 mo	Median OS NR, PFS NR, 58% PR, no CR, patterns of failure and impact of histology NR
Blumenschein et al. [21]	Multi-centre phase II, US, n = 87	Inoperable, Zubrod 0-1	Stage IIIA or B, weight loss < 5%, FEV1 ≥ 1,2 l	63 Gy in 35 fractions of 1.8 Gy, ENI to ipsilateral hilar and mediastinal nodes (45 Gy)	Cetuximab SR week 1-17, weekly carboplatin/paclitaxel during RT followed by 2 cycles consolidation carboplatin/paclitaxel	Median 22 mo	Median OS 22.7 mo, median time to progression around 14-15 mo, 29% CR, 33% PR, patterns of failure and impact of histology NR
Govindan et al. [23]	Multi-centre phase II, randomised, US, n = 101	Inoperable, ECOG 0-1, one of two trials with mandatory PET	Stage IIIA or B, no pleural effusion, weight loss ≤ 10%	70 Gy in 35 fractions of 2 Gy, no ENI	Cetuximab SR (7 weeks) plus 4 cycles carboplatin/pemetrexed vs. same CTx without cetuximab (n = 48), afterwards 4 cycles of pemetrexed	Median 32 mo	Median OS 25.2 mo*, median failure-free survival 12.3 mo, 4% CR, 68% PR, patterns of failure NR, survival not influenced by histology

RT: radiotherapy; IMRT: intensity-modulated radiotherapy; CTx: chemotherapy; KPS: Karnofsky performance status; ECOG: Eastern Cooperative Oncology Group performance status; WHO: World Health Organisation performance status; FEV1: forced expiratory volume 1; V20: lung volume receiving 20 Gy; ENI: elective nodal irradiation; Cetuximab SR: standard regimen with 400 mg/m^2 ^given i.v. on day 1 and 250 mg/m^2 ^on days 8, 15, 22, 29, 36 and 43; OS: overall survival; PFS: progression-free survival; PR and CR: partial and complete remission as per RECIST criteria; NR: not reported; PET: positron emission tomography

* all results relate to the cetuximab arm of the study

**Table 2:** Baseline characteristics and adverse events (AE)

Reference	Median age	Performance status	Stage	Weight loss	Histology	Adverse events (AE)
Jensen et al. [16]	71 years (57-82)	NR	II: 7% IIIA: 57% IIIB: 37%	Weight loss < 5% required	Adenocarcinoma: 33% Squamous: 57% Other or unknown: 10%	10% died before completing protocol treatment (death unlikely related to treatment), 50% had ≥ grade 3 non-hematologic AE
Jatoi et al. [15]	77 years (60-87)	0: 22% 1: 57% 2: 21%	IIIA: 59% IIIB: 41%	NR	Adenocarcinoma: 38% Squamous: 43% Other or unspecified: 19%	No treatment-related deaths, 9% stopped early because of AE, 53% had at least one AE ≥ grade 3
Hallqvist et al. [19]	62 years (42-81)	0: 62% 1: 38%	IIIA: 37% IIIB: 63%	> 5%: 37%	Adenocarcinoma: 49% Squamous: 39% Unspecified: 12%	One pneumonitis-related death, 18% did not complete cetuximab and 11% received < 68 Gy, 43% AE ≥ grade 3
Hughes et al. [20]	67.5 years (58-76)	0: 42% 1: 58%	IIIA: 33% IIIB: 50% IV: 17%	NR	Adenocarcinoma: 33% Squamous: 50% Other or unspecified: 17%	One pneumonia-related death, 17% did not complete cetuximab, 17% AE ≥ grade 3
Blumenschein et al. [21]	64 years (42-85)	0: 47% 1: 53%	IIIA: 46% IIIB: 54%	Weight loss < 5% required	NR	6 treatment-related deaths, 20% did not complete RT and concurrent cetuximab, 68% had ≥ grade 3 non-hematologic AE
Govindan et al. [23]*	66 years (32-81)	0: 34% 1: 66%	IIIA: 53% IIIB: 47%	Weight loss ≤ 10% required	Adenocarcinoma: 42% Squamous: 34% Other or unspecified: 25%	3 treatment-related deaths, 19% did not complete 4 cycles of CTx and 7 weeks of cetuximab, 62% had ≥ grade 3 non-hematologic AE and 70% ≥ grade 3 hematologic AE

NR: not reported; RT: radiotherapy

*data shown are for patients in the cetuximab arm of this randomised study

### Cetuximab and chemoradiation: induction chemotherapy only

Two phase II studies have been published, as shown in Tables 1 and 2[19,20]. Both included patients with inoperable stage III disease and good performance status. In the Swedish study, 2 cycles of induction cisplatin/docetaxel were administered [19]. The small UK study with a median of 3 cycles included patients treated with different regimens [20]. In both studies cetuximab was given concomitant to RT (total dose 64 and 68 Gy, respectively). ENI was administered to selected patients in the UK trial only. Serial computed tomography (CT) revealed that the UK trial included some patients with actual stage IV disease. Only the Swedish trial reported detailed outcome data. Median survival was 17 months, i.e. comparable to the results of the two trials without any chemotherapy [15,16] or other studies of sequential chemotherapy and RT without cetuximab [18]. Toxicity and compliance were also comparable to these aforementioned studies. In the absence of randomised trials, these sparse data do not create enthusiasm about further studies of comparable approaches. It appears more attractive to put resources into trials of the other two categories reviewed here.

### Cetuximab and chemoradiation: concomitant chemotherapy

The Radiation Therapy Oncology Group (RTOG) has recently published a phase II study of chemoradiotherapy with carboplatin and paclitaxel plus cetuximab in patients with stage III NSCLC [21]. From the loading dose of cetuximab to the end of the consolidation phase, 17 weekly treatments were administered. The radiation dose was 63 Gy in 35 fractions and chemotherapy was administered concurrently and in the consolidation phase. The authors concluded that treatment was feasible and survival longer than any previously reported by the RTOG. Median survival was 22.7 months. However, median time to progression was approximately 14-15 months (estimated from the graph). In other words, early cancer progression continues to be common. Moreover, RTOG trial 0117 reported median survival of 21.6 months in 44 patients with inoperable stage III NSCLC treated with 74 Gy and concomitant carboplatin/paclitaxel [22]. Median survival was numerically longer in the cetuximab trial but the absolute difference was 1 month. The second trial in this category was also completed in the US [23]. Several important differences exist (mandatory PET, higher radiation dose of 70 Gy, only 7 weeks of cetuximab concomitant to RT, chemotherapy with carboplatin and pemetrexed). Median survival was 25.2 months and failure-free survival 12.3 months. Given the large differences in study treatment, direct comparison appears difficult. It is important to mention that the two trials reviewed in this category reported higher rates of treatment-related deaths and adverse events than all the other studies where no concomitant chemotherapy was given. However, they also reported encouraging survival results, which led the investigators to initiate a confirmatory intergroup phase III trial that will clarify the role of additional cetuximab in this setting (RTOG 0617). Compared to other phase II studies without cetuximab, the survival results found in the two US trials are good but not exceptional. Table 3 contains a brief summary of relevant data, including selected arms from randomised trials, illustrating the possibility of impressive survival outcomes with quite different approaches. As mentioned previously, several sources of bias make comparisons between all these studies unreliable. Therefore, the present overview can not provide definitive recommendations.

**Table 3:** Results of other recent chemoradiation trials, unresectable stage III (without cetuximab)

Author, patient number	Treatment	Median survival (PFS)
Bepler et al. [41], 39	2 cycles induction carboplatin and gemcitabine, RT up to 74 Gy (mean 70) with weekly carboplatin and paclitaxel	22.7 mo (14.3 mo)

Socinski et al. [42], 43	2 cycles induction carboplatin and paclitaxel, RT 74 Gy with weekly carboplatin and paclitaxel	24.3 mo

Krzakowski et al. [43], 54	2 cycles induction cisplatin and vinorelbine, RT 66 Gy with 2 cycles cisplatin and vinorelbine	23.4 mo (12.5 mo)

Sejpal et al. [44], 62	Weekly carboplatin and paclitaxel, RT median 74 Gy with protons	24.4 mo

Segawa et al. [45], 99	RT 60 Gy with 4 cycles of concomitant cisplatin and docetaxel	26.8 mo (13.4 mo)

Cho et al. [46], 49	Weekly carboplatin and paclitaxel, RT 60 Gy in 25 fractions	28.1 mo (13.7 mo)

Gandara et al. [47], 83	RT 61 Gy with 2 cycles of concomitant cisplatin and etoposide, consolidation docetaxel	26 mo (16 mo)

RT: radiotherapy; PFS: progression-free survival

## Discussion

Historically, the added value of cetuximab has been proven in a pivotal head and neck cancer radiotherapy trial, which did not include cytotoxic chemotherapy [14]. This trial confirmed preclinical results obtained in different in vitro and in vivo models [9-13]. The NSCLC studies reviewed in the present article suggest that conventional fractionated RT (3-D conformal or intensity-modulated) to a maximum dose of 70 Gy can safely be combined with cetuximab. With additional concomitant chemotherapy, toxicity increases to the high degree that has been observed in several studies of simultaneous chemoradiation without cetuximab [1,4,6]. Over 60% of patients developed ≥ grade 3 non-hematologic adverse events and comparable figures were reported for ≥ grade 3 hematologic adverse events, which contributed to the fact that approximately 20% of patients were unable to complete treatment [21,23]. Importantly, no randomised head-to-head comparison of any of the 3 strategies reviewed here (RT plus cetuximab, induction chemotherapy followed by RT plus cetuximab, concomitant chemoradiation plus cetuximab) has yet been published. Therefore, both toxicity and efficacy results must be interpreted with caution. Non-randomised head and neck cancer studies suggested promising efficacy of combined cetuximab, RT and chemotherapy [24-26]. However, the phase III trial RTOG 0522 could not confirm improved progression-free or overall survival when cetuximab was added to RT with cisplatin [27]. Conflicting results have also been reported for rectal cancer [28].

This review does not attempt to provide treatment recommendations. Its purpose is to contribute to the development of future, large-scale prospective studies. Such studies seem warranted mainly in two settings 1) RT plus cetuximab in patients who can not tolerate chemoradiation, and 2) concomitant chemoradiation plus cetuximab in patients who qualify for such aggressive regimens. Regarding the latter approach, a randomised intergroup study based on the results of the RTOG trial [21] is under way. A question that will not be answered in this trial is the optimal duration of cetuximab treatment, provided there is a definitive benefit from this drug. As reviewed here, some groups chose to limit drug administration to the concomitant phase with RT while others continued cetuximab for longer durations. Given the cost of such treatment, the added value of extended cetuximab therapy must be proven in appropriate randomised settings. Other well recognised areas of controversy that apply to all NSCLC RT strategies including the cetuximab trials, are the role of ENI [7,8,29], PET for staging and treatment planning [8,30-32], radiation dose escalation [8,18,22] and consolidation chemotherapy after chemoradiation [2,33]. The variations in the 6 studies reviewed here nicely illustrate the uncertainties around these issues. None of these studies used accelerated RT regimens or hypofractionation in combination with cetuximab. However, altered fractionation RT resulting in shorter overall treatment time is one of the possibilities to improve NSCLC outcomes [34,35].

Histology has gained increasing importance for the choice of NSCLC systemic therapy but had no significant influence on survival in the 3 cetuximab plus RT studies that looked at this parameter [16,19,23]. Whether these studies truly suggest that inclusion of any histologic type of NSCLC into future trials should be considered remains an open question, given their limited sample size and statistical power. However, individually tailored treatment has the potential to improve cost-effectiveness and spare patients from unnecessary toxicity. Unfortunately, at present no established biomarker or histology feature has gained widespread acceptance. A recent analysis of a phase III study (BMS099; taxane/carboplatin with or without cetuximab; no radiotherapy) where tumour samples from 225 patients were examined, did not find significant associations between KRAS and EGFR and various outcome parameters [36]. Also in the phase III FLEX study (cisplatin/vinorelbine with or without cetuximab; no radiotherapy) biomarkers (KRAS, EGFR, PTEN) did not predict treatment efficacy [37]. On the basis of all available data, it is not justified to exclude molecular subgroups of NSCLC from future RT and cetuximab studies. However, it is important to conduct additional biomarker analyses in these future studies. If EGFR inhibition increases the clinical efficacy of RT for stage III NSCLC, cetuximab might not be the only agent exerting this effect. Preliminary experience with tyrosine kinase inhibitors and thoracic RT has been published [38,39]. However, these small non-randomised studies suffer from the same limitations as those reviewed here and have not provided definitive data. Because the presence of EGFR mutations in general is predictive of responsiveness to EGFR tyrosine kinase inhibitors, adenocarcinoma or NSCLC not otherwise specified should be tested for such alterations [40].

## Conclusions

The results of the 6 published clinical trials (none of them was a phase III study) suggest that larger randomised trials are warranted, primarily addressing the role of cetuximab with RT alone in patients unfit for chemoradiation and combined chemoradiation plus cetuximab in prognostically better patients. Correlative biomarker studies should be part of these research efforts.

## Competing interests

The authors declare that they have no competing interests.

## Authors' contributions

CN and NA participated in the design of the study. CN, AP and AD performed the literature search, extracted relevant articles and drafted the manuscript. All authors read and approved the final manuscript.

## References

[B1] Baas P Belderbos JS van den Heuvel M Chemoradiation therapy in nonsmall cell lung cancer Curr Opin Oncol 2011 23 140 149 10.1097/CCO.0b013e328341eed6 21178617

[B2] Thatcher N Heighway J Maintenance and consolidation therapy in patients with unresectable stage III/IV non-small cell lung cancer Oncologist 2010 15 1034 1042 10.1634/theoncologist.2009-0292 20930098 PMC3227898

[B3] Anderson CS Curran WJ Combined modality therapy for stage III non-small-cell lung cancer Semin Radiat Oncol 2010 20 186 191 10.1016/j.semradonc.2010.01.007 20685581

[B4] O'Rourke N Roqué I Figuls M Farré Bernadó N Macbeth F Concurrent chemoradiotherapy in non-small cell lung cancer Cochrane Database Syst Rev 2010 CD002140 10.1002/14651858.CD002140.pub3 PMC12584098 20556756

[B5] Wurstbauer K Weise H Deutschmann H Kopp P Merz F Studnicka M Nairz O Sedlmayer F Non-small cell lung cancer in stages I-IIIB: Long-term results of definitive radiotherapy with doses ≥ 80 Gy in standard fractionation Strahlenther Onkol 2010 186 551 557 10.1007/s00066-010-2108-3 20936459

[B6] Machtay M Bae K Movsas B Paulus R Gore EM Komaki R Albain K Sause WT Curran WJ Higher biologically effective dose of radiotherapy is associated with improved outcomes for locally advanced non-small cell lung carcinoma treated with chemoradiation: An analysis of the Radiation Therapy Oncology Group Int J Radiat Oncol Biol Phys 2010 epub 10.1016/j.ijrobp.2010.09.004 PMC5764542 20980108

[B7] Guckenberger M Wilbert J Richter A Baier K Flentje M Potential of adaptive radiotherapy to escalate the radiation dose in combined radiochemotherapy for locally advanced non-small cell lung cancer Int J Radiat Oncol Biol Phys 2011 79 901 908 10.1016/j.ijrobp.2010.04.050 20708850

[B8] De Ruysscher D Faivre-Finn C Nestle U Hurkmans CW Le Pechoux C Price A Senan S European Organisation for Research and Treatment of Cancer recommendations for planning and delivery of high-dose, high-precision radiotherapy for lung cancer J Clin Oncol 2010 28 5301 5310 10.1200/JCO.2010.30.3271 21079134

[B9] Andratschke NH Dittmann KH Mason KA Fan Z Liao Z Komaki R Ang KK Milas L Epidermal growth factor receptor as a target to improve treatment of lung cancer Clin Lung Cancer 2004 5 340 352 10.3816/CLC.2004.n.012 15217533

[B10] Wang M Morsbach F Sander D Gheorghiu L Nanda A Benes CH Kriegs M Krause M Dikomey E Baumann M Dahm-Daphi J Settleman JE Willers H EGF receptor inhibition radiosensitizes NSCLC cells by inducing senescence in cells sustaining DNA double-strand breaks Cancer Res 2011 epub 10.1158/0008-5472.CAN-11-0213 PMC3185115 21852385

[B11] Raben D Helfrich B Chan DC Ciardiello F Zhao L Franklin W Baron AE Zeng C Johnson TK Bunn PA Jr The effects of cetuximab alone and in combination with radiation and/or chemotherapy in lung cancer Clin Cancer Res 2005 11 795 805 15701870

[B12] Milas L Fan Z Andratschke NH Ang KK Epidermal growth factor receptor and tumor response to radiation: in vivo preclinical studies Int J Radiat Oncol Biol Phys 2004 58 966 971 10.1016/j.ijrobp.2003.08.035 14967457

[B13] Nasu S Ang KK Fan Z Milas L C225 antiepidermal growth factor receptor antibody enhances tumor radiocurability Int J Radiat Oncol Biol Phys 2001 51 474 477 10.1016/S0360-3016(01)01671-6 11567823

[B14] Bonner JA Harari PM Giralt J Cohen RB Jones CU Sur RK Raben D Baselga J Spencer SA Zhu J Youssoufian H Rowinsky EK Ang KK Radiotherapy plus cetuximab for locoregionally advanced head and neck cancer: 5-year survival data from a phase 3 randomised trial, and relation between cetuximab-induced rash and survival Lancet Oncol 2010 11 21 28 10.1016/S1470-2045(09)70311-0 19897418

[B15] Jatoi A Schild SE Foster N Henning GT Dornfeld KJ Flynn PJ Fitch TR Dakhil SR Rowland KM Stella PJ Soori GS Adjei AA A phase II study of cetuximab and radiation in elderly and/or poor performance status patients with locally advanced non-small-cell lung cancer (N0422) Ann Oncol 2010 21 2040 2044 10.1093/annonc/mdq075 20570832 PMC2980935

[B16] Jensen AD Münter MW Bischoff HG Haselmann R Haberkorn U Huber PE Thomas M Debus J Herfarth KK Combined treatment of nonsmall cell lung cancer stage III with intensity-modulated radiotherapy and cetuximab: The NEAR trial Cancer 2011 117 2986 2994 10.1002/cncr.25888 21264838

[B17] Jeremić B Miličić B Milisavljevic S Clinical prognostic factors in patients with locally advanced (stage III) nonsmall cell lung cancer treated with hyperfractionated radiation therapy with and without concurrent chemotherapy: single-institution experience in 600 patients Cancer 2011 117 2995 3003 10.1002/cncr.25910 21692056

[B18] Wang L Correa CR Zhao L Hayman J Kalemkerian GP Lyons S Cease K Brenner D Kong FM The effect of radiation dose and chemotherapy on overall survival in 237 patients with stage III non-small-cell lung cancer Int J Radiat Oncol Biol Phys 2009 73 1383 1390 10.1016/j.ijrobp.2008.06.1935 18929449 PMC3381887

[B19] Hallqvist A Wagenius G Rylander H Brodin O Holmberg E Löden B Ewers SB Bergström S Wichardt-Johansson G Nilsson K Ekberg L Sederholm C Nyman J Concurrent cetuximab and radiotherapy after docetaxel-cisplatin induction chemotherapy in stage III NSCLC: Satellite - A phase II study from the Swedish Lung Cancer Study Group Lung Cancer 2011 71 166 172 10.1016/j.lungcan.2010.05.011 20541833

[B20] Hughes S Liong J Miah A Ahmad S Leslie M Harper P Prendiville J Shamash J Subramaniam R Gaya A Spicer J Landau D A brief report on the safety study of induction chemotherapy followed by synchronous radiotherapy and cetuximab in stage III non-small cell lung cancer (NSCLC): SCRATCH study J Thorac Oncol 2008 3 648 651 10.1097/JTO.0b013e3181757a60 18520806

[B21] Blumenschein GR Jr Paulus R Curran WJ Robert F Fossella F Werner-Wasik M Herbst RS Doescher PO Choy H Komaki R Phase II study of cetuximab in combination with chemoradiation in patients with stage IIIA/B non-small-cell lung cancer: RTOG 0324 J Clin Oncol 2011 29 2312 2318 10.1200/JCO.2010.31.7875 21555682 PMC3107747

[B22] Bradley JD Bae K Graham MV Byhardt R Govindan R Fowler J Purdy JA Michalski JM Gore E Choy H Primary analysis of the phase II component of a phase I/II dose intensification study using three-dimensional conformal radiation therapy and concurrent chemotherapy for patients with inoperable non-small-cell lung cancer: RTOG 0117 J Clin Oncol 2010 28 2475 2480 10.1200/JCO.2009.27.1205 20368547 PMC2881726

[B23] Govindan R Bogart J Stinchcombe T Wang X Hodgson L Kratzke R Garst J Brotherton T Vokes EE Randomized phase II study of pemetrexed, carboplatin, and thoracic radiation with or without cetuximab in patients with locally advanced unresectable non-small-cell lung cancer: Cancer and Leukemia Group B Trial 30407 J Clin Oncol 2011 29 3120 3125 10.1200/JCO.2010.33.4979 21747084 PMC3157978

[B24] Argiris A Heron DE Smith RP Kim S Gibson MK Lai SY Branstetter BF Posluszny DM Wang L Seethala RR Dacic S Gooding W Grandis JR Johnson JT Ferris RL Induction docetaxel, cisplatin, and cetuximab followed by concurrent radiotherapy, cisplatin, and cetuximab and maintenance cetuximab in patients with locally advanced head and neck cancer J Clin Oncol 2010 28 5294 5300 10.1200/JCO.2010.30.6423 21079141 PMC3018361

[B25] Kao J Genden EM Gupta V Policarpio EL Burri RJ Rivera M Gurudutt V Som PM Teng M Packer SH Phase 2 trial of concurrent 5-fluorouracil, hydroxyurea, cetuximab, and hyperfractionated intensity-modulated radiation therapy for locally advanced head and neck cancer Cancer 2011 117 318 326 10.1002/cncr.25374 20830768

[B26] Merlano M Russi E Benasso M Corvò R Colantonio I Vigna-Taglianti R Vigo V Bacigalupo A Numico G Crosetto N Gasco M Lo Nigro C Vitiello R Violante S Garrone O Cisplatin-based chemoradiation plus cetuximab in locally advanced head and neck cancer: a phase II clinical study Ann Oncol 2011 22 712 717 10.1093/annonc/mdq412 20810547

[B27] Ang KK Zhang QE Rosenthal DI Nguyen-Tan P Sherman EJ Weber RS Galvin JM Schwartz DL El-Naggar AK Gillison ML Jordan R List MA Konski AA Thorstad WL Trotti A Beitler JJ Garden AS Spanos WJ Yom SS Axelrod SS A randomized phase III trial (RTOG 0522) of concurrent accelerated radiation plus cisplatin with or without cetuximab for stage III-IV head and neck squamous cell carcinomas (HNC) J Clin Oncol 2011 29 suppl abstract 5500 10.1200/JCO.2013.53.5633 PMC4162493 25154822

[B28] Hu-Lieskovan S Vallbohmer D Zhang W Yang D Pohl A Labonte MJ Grimminger PP Hölscher AH Semrau R Arnold D Dellas K Debucquoy A Haustermans K Machiels JP Sempoux C Rödel C Bracko M Velenik V Lenz HJ EGF61 polymorphism predicts complete pathologic response to cetuximab-based chemoradiation independent of KRAS status in locally advanced rectal cancer patients Clin Cancer Res 2011 17 5161 5169 10.1158/1078-0432.CCR-10-2666 21673069 PMC3149732

[B29] Sulman EP Komaki R Klopp AH Cox JD Chang JY Exclusion of elective nodal irradiation is associated with minimal elective nodal failure in non-small cell lung cancer Radiat Oncol 2009 4 5 10.1186/1748-717X-4-5 19183471 PMC2651897

[B30] van Loon J van Baardwijk A Boersma L Ollers M Lambin P De Ruysscher D Therapeutic implications of molecular imaging with PET in the combined modality treatment of lung cancer Cancer Treat Rev 2011 37 331 343 10.1016/j.ctrv.2011.01.005 21320756

[B31] Lammering G De Ruysscher D van Baardwijk A Baumert BG Borger J Lutgens L van den Ende P Ollers M Lambin P The use of FDG-PET to target tumors by radiotherapy Strahlenther Onkol 2010 186 471 481 10.1007/s00066-010-2150-1 20814658

[B32] Sharma N Neumann D Macklis R The impact of functional imaging on radiation medicine Radiat Oncol 2008 3 25 10.1186/1748-717X-3-25 18793395 PMC2553402

[B33] Cheruvu P Metcalfe SK Metcalfe J Chen Y Okunieff P Milano MT Comparison of outcomes in patients with stage III versus limited stage IV non-small cell lung cancer Radiat Oncol 2011 6 80 10.1186/1748-717X-6-80 21718501 PMC3141527

[B34] Wurstbauer K Deutschmann H Kopp P Kranzinger M Merz F Nairz O Studnicka M Sedlmayer F Nonresected non-small-cell lung cancer in Stages I through IIIB: accelerated, twice-daily, high-dose radiotherapy - a prospective Phase I/II trial with long-term follow-up Int J Radiat Oncol Biol Phys 2010 77 1345 1351 10.1016/j.ijrobp.2009.06.060 19910140

[B35] Hatton MQ Martin JE Continuous hyperfractionated accelerated radiotherapy (CHART) and non-conventionally fractionated radiotherapy in the treatment of non-small cell lung cancer: a review and consideration of future directions Clin Oncol (R Coll Radiol) 2010 22 356 364 10.1016/j.clon.2010.03.010 20399629

[B36] Khambata-Ford S Harbison CT Hart LL Awad M Xu LA Horak CE Dakhil S Hermann RC Lynch TJ Weber MR Analysis of potential predictive markers of cetuximab benefit in BMS099, a phase III study of cetuximab and first-line taxane/carboplatin in advanced non-small-cell lung cancer J Clin Oncol 2010 28 918 927 10.1200/JCO.2009.25.2890 20100958

[B37] O'Byrne KJ Gatzemeier U Bondarenko I Barrios C Eschbach C Martens UM Hotko Y Kortsik C Paz-Ares L von Pawel J Ramlau R Roh JK Yu CT Stroh C Celik I Schueler A Pirker R Molecular biomarkers in non-small-cell lung cancer: a retrospective analysis of data from the phase 3 FLEX study Lancet Oncol 2011 12 795 805 10.1016/S1470-2045(11)70189-9 21782507

[B38] Wang J Xia TY Wang YJ Li HQ Li P Wang JD Chang DS Liu LY Di YP Wang X Wu WZ Prospective study of epidermal growth factor receptor tyrosine kinase inhibitors concurrent with individualized radiotherapy for patients with locally advanced or metastatic non-small-cell lung cancer Int J Radiat Oncol Biol Phys 2011 81 e59 65 10.1016/j.ijrobp.2010.12.035 21345607

[B39] Ready N Jänne PA Bogart J Dipetrillo T Garst J Graziano S Gu L Wang X Green MR Vokes EE Chemoradiotherapy and gefitinib in stage III non-small cell lung cancer with epidermal growth factor receptor and KRAS mutation analysis: cancer and leukemia group B (CALGB) 30106, a CALGB-stratified phase II trial J Thorac Oncol 2010 5 1382 1390 10.1097/JTO.0b013e3181eba657 20686428

[B40] Travis WD Brambilla E Noguchi M Nicholson AG Geisinger KR Yatabe Y Beer DG Powell CA Riely GJ Van Schil PE Garg K Austin JH Asamura H Rusch VW Hirsch FR Scagliotti G Mitsudomi T Huber RM Ishikawa Y Jett J Sanchez-Cespedes M Sculier JP Takahashi T Tsuboi M Vansteenkiste J Wistuba I Yang PC Aberle D Brambilla C Flieder D Franklin W Gazdar A Gould M Hasleton P Henderson D Johnson B Johnson D Kerr K Kuriyama K Lee JS Miller VA Petersen I Roggli V Rosell R Saijo N Thunnissen E Tsao M Yankelewitz D International association for the study of lung cancer/american thoracic society/european respiratory society international multidisciplinary classification of lung adenocarcinoma J Thorac Oncol 2011 6 244 285 10.1097/JTO.0b013e318206a221 21252716 PMC4513953

[B41] Bepler G Dilling TJ Wagner H Hazelton T Williams C Chen DT Greenberg H Walsh F Simon G Tanvetyanon T Chiappori A Haura E Stevens C Phase II trial of induction gemcitabine and carboplatin followed by conformal thoracic radiation to 74 Gy with weekly paclitaxel and carboplatin in unresectable stage III non-small cell lung cancer J Thorac Oncol 2011 6 553 558 10.1097/JTO.0b013e31820b8d88 21289520 PMC3839293

[B42] Socinski MA Blackstock AW Bogart JA Wang X Munley M Rosenman J Gu L Masters GA Ungaro P Sleeper A Green M Miller AA Vokes EE Randomized phase II trial of induction chemotherapy followed by concurrent chemotherapy and dose-escalated thoracic conformal radiotherapy (74 Gy) in stage III non-small-cell lung cancer: CALGB 30105 J Clin Oncol 2008 26 2457 2463 10.1200/JCO.2007.14.7371 18487565

[B43] Krzakowski M Provencio M Utracka-Hutka B Villa E Codes M Kuten A Henke M Lopez M Bell D Biti G Merimsky O Beorchia A Riggi M Caux NR Pouget JC Dubray B David P Oral vinorelbine and cisplatin as induction chemotherapy and concomitant chemo-radiotherapy in stage III non-small cell lung cancer: final results of an international phase II trial J Thorac Oncol 2008 3 994 1002 10.1097/JTO.0b013e31818396cb 18758302

[B44] Sejpal S Komaki R Tsao A Chang JY Liao Z Wei X Allen PK Lu C Gillin M Cox JD Early findings on toxicity of proton beam therapy with concurrent chemotherapy for nonsmall cell lung cancer Cancer 2011 117 3004 3013 10.1002/cncr.25848 21264827

[B45] Segawa Y Kiura K Takigawa N Kamei H Harita S Hiraki S Watanabe Y Yonei T Ueoka H Takata I Hotta K Hiraki A Tabata M Matsuo K Tanimoto M Phase III trial comparing docetaxel and cisplatin combination chemotherapy with mitomycin, vindesine, and cisplatin combination chemotherapy with concurrent thoracic radiotherapy in locally advanced non-small-cell lung cancer: OLCSG 0007 J Clin Oncol 2010 28 3299 3306 10.1200/JCO.2009.24.7577 20530281

[B46] Cho KH Ahn SJ Pyo HR Kim KS Kim YC Moon SH Han JY Kim HT Koom WS Lee JS A phase II study of synchronous three-dimensional conformal boost to the gross tumor volume for patients with unresectable stage III non-small-cell lung cancer: results of Korean Radiation Oncology Group 0301 study Int J Radiat Oncol Biol Phys 2009 74 1397 1404 10.1016/j.ijrobp.2008.10.020 19117690

[B47] Gandara DR Chansky K Albain KS Gaspar LE Lara PN Jr Kelly K Crowley J Livingston R Long-term survival with concurrent chemoradiation therapy followed by consolidation docetaxel in stage IIIB non-small-cell lung cancer: a phase II Southwest Oncology Group study (S9504) Clin Lung Cancer 2005 8 116 121 10.3816/CLC.2006.n.039 17026812

